# Predicting attitudes toward ambiguity using natural language processing on free descriptions for open-ended question measurements

**DOI:** 10.1038/s41598-024-59118-z

**Published:** 2024-04-09

**Authors:** Jimpei Hitsuwari, Hirohito Okano, Michio Nomura

**Affiliations:** 1 Graduate School of Education, Kyoto University, Kyoto, Japan; 2 Japan Society for the Promotion of Science, Tokyo, Japan

**Keywords:** Psychology, Human behaviour, Computer science

## Abstract

Individual traits and reactions to ambiguity differ and are conceptualized in terms of an individual’s attitudes toward ambiguity or ambiguity tolerance. The development of natural language processing technology has made it possible to measure mental states and reactions through open-ended questions, rather than predefined numerical rating scales, which have traditionally been the dominant method in psychological research. This study presented three ambiguity-related situations and responses collected online from 591 participants in an open-ended format. After the analysis with bidirectional encoder representations from transformers, correlations were calculated using scores from the numerical evaluation by conventional questionnaire, and a significant moderate positive correlation was found. Therefore, this study found that attitudes toward ambiguity can be measured using an open-ended response method of reporting everyday life states. It is a novel methodology that can be expanded to other scales in psychology and can potentially be used in educational and clinical situations where participants can be asked to respond with minimal burden.

## Introduction

The ambiguous situations faced in the volatility, uncertainty, complexity, and ambiguity (VUCA) era are diverse, with individual differences in attitudes toward these ambiguous situations^1^. To measure individual differences, Lauriola et al.^2^ developed the Multidimensional Attitude toward Ambiguity Scale (MAAS) based on the Ambiguity Tolerance Scale, which measures individuals’ tolerance degree toward ambiguous situations. This scale has been validated for construct validity and internal reliability^2^. The MAAS is utilized globally, with Japanese^3^ and Swedish versions^4^ also being developed. It has been used in numerous behavioral experiments and psychological surveys^5,6^.

However, responding to a predefined numerical rating scale is not necessarily the optimal method to capture complex mental states and personality traits (People do not usually answer or express their states and emotions on a yes or no or 1–7 point scale, and most often use natural language.; for review, see^7^). Considering the recent popularity of ChatGPT, the development of large language models has made it possible to measure psychological states based on natural language, which was quite challenging in the past. For example, in Kjell et al.’s study, participants had to answer the question, “Overall, in your life, are you satisfied or not?”^8^. They examined the correlation between the values calculated by bidirectional encoder representations from transformers (BERT), a large language model, and the scores of the Satisfaction with Life Scale (SWLS)^9^, which has been conventionally used to measure life satisfaction. The BERT regression model transforms the participant’s free text into a multidimensional vector and uses that vector representation to predict the individual’s questionnaire score. The results indicated *r* = 0.74, implying that life satisfaction can be accurately measured using open-ended responses. In another study^10^, BERT was used to predict the Big Five personality traits based on user comments and posts comprising fiction (e.g., short stories) in a novel-writing community on Reddit (a bulletin board social site). The results indicated an average performance of *r* = 0.33, suggesting that personality can be predicted using free text. The present study asked participants to respond to open-ended questions in three situations (see below in the Method section) involving ambiguity (from the MAAS subscale), and the obtained texts were analyzed. The study aimed to determine the extent to which the survey methods consisting of free-text and natural language processing (NLP) predicted ambiguity tolerance in comparison to conventional numerical scores. Additionally, this study examined whether the texts answered from the respective MAAS subscales could discriminate between the respective subscales answered with numerical values.

## Methods

This study was approved by the Ethics Committee of the Graduate School of Education at Kyoto University (CPE-571) and conducted in accordance with relevant guidelines and regulations. We obtained informed consent from the study participants before their participation.

### Participants

A total of 600 native English speakers of British nationality (the language used in most of the previous studies^8^ is English, so we targeted British nationals referring to those NLP studies) were recruited using an online survey platform Prolific (https://www.prolific.com/). Nine were excluded because of duplicate IP addresses, extremely short response times (less than 255 s), and attention-checking errors, resulting in 591 participants (M_age_ = 43.35, SD = 14.44, 325 males, 255 females, 11 others) for the final analyses. A question for the attention check (For this question, select “5. I mildly agree”) was added to MSTAT II (detailed in Procedure section) to exclude participants who selected anything other than the required answer. They were paid￡0.6 as a reward for their participation.

### Procedure

The participants provided open-ended responses to three ambiguous situations. The three situations correspond to the three factors of the MAAS: “How do you typically react when you are uncertain about the responsibilities of a job? (Discomfort with Ambiguity; DA),” “How do you typically react when ambiguous words like ‘probably,’ ‘approximately,’ or ‘perhaps’ are used? (Absolutism; AB),” and “How do you typically react when you are in situations which can be interpreted in more than one way? (Need for Complexity and Novelty; NC)” The responses were required to have at least 100 characters (approximately 20 words), and at least 45 s had to pass before answering the next question. Subsequently, participants responded to a questionnaire containing the MAAS and the Multiple Stimulus Types Ambiguity Tolerance Scale-II (MSTAT-II)^11^. The MSTAT-II is a general measure of ambiguity tolerance and was employed to determine whether it could predict this scale score from the three situations created from the MAAS (usually, in MAAS, the average of each subscale score is calculated but not the overall score). Finally, respondents’ demographic data (sex, age, nationality, and education) were collected. Descriptive statistics from the MAAS and MSTAT-II and examples of open-ended responses obtained from the three texts are presented in Table 1.

**Table 1:** MAAS and MSTAT descriptive statistics and examples of free-text responses from the three situations.

	M	SD	Examples
DA	4.48	1.12	I get into a bit of a panic, try to work out how to do it on my own, and if I am still unable to, I go to someone with my tail between my legs
			Wait and see; I do not get stressed, I seek to clarify exactly what the responsibilities will be and, if needed, double-clarify
MA	3.35	1.18	I do not mind them if it is just occasionally, but it can be annoying when they are used in every other sentence
			I would feel slightly distrustful and cautious. I have learned never to take assurances at face value. It depends on who is using such language
NC	4.03	1.18	I always take my time, thinking through the options and how my actions could be interpreted by the people around me
			I react confused, I find it difficult to have to pick a certain path because I always question whether I have chosen correctly
MSTAT	4.13	0.89	

### Analysis

The model for predicting the questionnaire scores was developed by fine-tuning the pre-trained BERT-base-cased model (https://huggingface.co/bert-base-cased). Closed models like ChatGPT raise scientific reproducibility and ethical concerns, as the precise architecture and training data are not disclosed, and updates are made without revealing the differences^7^. Therefore, for this study, a more open model, BERT, was used. Regarding hyperparameter selection during fine-tuning and final model evaluation, five-fold nested cross-validation (nested CV) was used. The nested CV has a low bias in estimation accuracy^12^ and is particularly effective for machine learning on small samples^13^. It allows obtaining an estimate of the model’s predictive accuracy, independent of the data used to build the model (see Supplementary Material for more information).

## Results

The correlation coefficients between the BERT-predicted and true values of the questionnaire scores when using free-text responses to the three open-ended questions were calculated (Table 2 presents the medians; see Supplementary Table 1 for the minimum and maximum values). Results indicated that text NC (*r* = 0.38, *p* < 0.001) and the text combining all three texts (*r* = 0.41, *p* < 0.001) moderately predicted the MSTAT-II scores, which measure general ambiguity tolerance. Additionally, texts from the DA (*r* = 0.28, *p* = 0.002), AB (*r* = 0.23, *p* = 0.01), and NC (*r* = 0.19, *p* = 0.04) were weakly correlated with their respective MAAS subscale scores.

**Table 2:** Median correlation coefficient between each text and each questionnaire score.

	DA	AB	NC	MSTAT
Text DA	0.28** [0.10, 0.44]	0.12 [− 0.07, 0.29]	0.10 [− 0.08, 0.28]	0.20* [0.02, 0.37]
Text AB	0.12 [− 0.06, 0.29]	0.23* [0.05, 0.40]	0.14 [− 0.04, 0.32]	0.06 [− 0.12, 0.24]
Text NC	0.26** [0.09, 0.42]	0.16† [− 0.02, 0.33]	0.19* [0.01, 0.36]	0.38*** [0.22, 0.53]
All texts	0.34*** [0.18, 0.49]	0.25** [0.07, 0.41]	0.28** [0.11, 0.44]	0.41*** [0.25, 0.55]

Text DA (Discomfort with Ambiguity), text AB (Absolutism), and text NC (Need for Complexity and Novelty) were calculated as free-text responses obtained from open-ended questions adopted from the DA, AB, and NC questions, respectively. The correlation coefficients in the table show the median correlation coefficients between the predicted values and true ones for the five test sets in the outer loop (n = 591/5 = 118 or 119). All texts were calculated by combining these three questions. *** *p* < 0.001, ** *p* < 0.01, * *p* < 0.05, † *p* < 0.10.

## Discussion

The findings of this study are novel as they indicate that even free text can predict psychological states and traits^8,10^ with regard to ambiguity.

Three questions were asked in this study; however, only one question from NC, “How do you typically react when you are in situations which can be interpreted in more than one way?” was moderately predictive. This question is more general than the other two questions and applies to various situations. This suggests that refining situation settings and how questions are asked may allow attitudes toward ambiguity to be measurable, even with only one open-ended response. The DA, AB, and NC texts showed weak but significant correlations with their respective scores. Future studies should consider making it possible to discriminate between subscales, for example, by devising how the questions are asked.

This survey method consisting of free-text and NLP will allow for the measuring of an individual’s personality in a more ecologically valid form; that is, an open-ended response method when expressing emotions and states in everyday life^8,10,14,15^. In Kjell et al.’s study^8^, questions aimed to examine overall life satisfaction, such as “Overall, in your life, are you satisfied or not?”; however, in this study, the question was constructed by specifying the situation and asking the respondent to imagine the situation, where “it can be interpreted in more than one way.” This allows the use of open-ended surveys that measure not only abstract concepts, such as life satisfaction, but also other personality traits and psychological states that are more specific.

While moderate correlation coefficients were observed, aligning with previous studies^10^, there is scope for further improvement in correlation by employing alternative language models (e.g., RoBERTa), a topic of interest for future studies. Consistent with previous studies, the results of this study are limited to English-language data. However, given the translation of the scale into various languages, efforts will be made to globally predict its scores in open-ended surveys in the future study. Both the MAAS and MSTAT-II used in this study were self-reported, and future research can attempt to predict a behavior (e.g., decision-making in ambiguous situations) based on participants’ open-ended responses and BERT scores.

In conclusion, this study successfully predicted attitudes toward ambiguity by NLP of open-ended responses using BERT. Through the utilization of these technologies, complex human minds can be measured in a way that is natural to the participants, with little concern that the content of the questionnaire items will influence participants’ cognitions. Academically, as the scale is translated into other languages, attempts can be made to predict its scores in open-ended surveys globally to increase its accuracy and discrimination to apply it to social surveys, education, clinical situations, among other spheres.

### Supplementary Information

Supplementary Information.

## Data Availability

All data and script are available online (https://osf.io/jza53/?view_only=dbdc4b4c82f94410aed7e5ccbb22a98d).
