# Supplementary material for: Predicting attitudes toward ambiguity using natural language processing on free descriptions for open-ended question measurements
Source: Sci Rep. 2024 Apr 9;14:8276. doi: 10.1038/s41598-024-59118-z (PMC11004121; doi:10.1038/s41598-024-59118-z)
Supplement: Supplementary file 1 — Supplementary Information. [file 41598_2024_59118_MOESM1_ESM.docx]

**Supplementary material**

The hyperparameters tested during fine-tuning were {2e-5, 3e-5, 4e-5, 5e-5} for the learning rate, {8, 16} for the batch size, and {1, 2, 3, 4, 5} for the number of epochs. The warm-up rate was set at 0.1. The model was constructed using the Transformers package in Python. The optimizer for training was AdamW, the loss function was the mean square error (MSE), and the accuracy measures for model evaluation was Pearson correlation coefficient between true value of the scale score and the predicted score from the BERT model.

The procedure for nested 5-fold cross-validation in this study was as follows:

First, the dataset was shuffled and divided into five sub-datasets. The subsequent process consisted of outer and inner loops.

**Outer loop**

Five sub-datasets were individually selected as test data, and the remaining four sub-datasets were used as model-development data. Using the development data, a model was developed according to the inner loop procedure as described below, and accuracy (Pearson correlation coefficient) was obtained by predicting the test data using the developed model. As five sub-datasets served as test data, this process was repeated five times. The median of the five obtained Pearson correlation coefficients was the final accuracy of the BERT prediction of the questionnaire scores.

**Inner loop**

The four sub-datasets that comprised the development data were individually selected as validation data, and the remaining three sub-datasets became training data. After fine-tuning the model for each combination of hyperparameters using the training data (resulting in 40 different models), the Pearson correlation coefficient was recorded when the validation data were predicted using each of the 40 models trained using each hyperparameter. As four sub-datasets served as validation data, this process was repeated four times. For each hyperparameter combination, the four Pearson correlation coefficients obtained within the inner loop were averaged, and the model was developed using all four subsets (the entirety of the building data) and the hyperparameter combination that achieved the highest average accuracy. Subsequently, the built model was tested using the test data in an outer loop as described earlier.

The median of the five Pearson coefficients (n = 118 or 119) obtained in the above outer loop was used to evaluate the accuracy of the constructed model. A test of no correlation of the median Pearson correlation coefficient was performed to obtain a *p*-value and 95% confidence interval. A prediction accuracy was considered significant when *p* < .05.

**Supplementary Table S1**

*Minimum and maximum correlation coefficients between each text and each questionnaire score*

|  | DA | | |  | AB | | |  | NC | | |  | MSTAT | | |
| --- | --- | --- | --- | --- | --- | --- | --- | --- | --- | --- | --- | --- | --- | --- | --- |
|  | Min | Median | Max |  | Min | Median | Max |  | Min | Median | Max |  | Min | Median | Max |
| Text DA | **.18** | **.28** | **.32** |  | .00 | .12 | **.19** |  | -.02 | .10 | **.19** |  | .09 | **.22** | **.36** |
| Text AB | .04 | .12 | **.22** |  | .08 | **.23** | **.31** |  | .10 | .14 | **.23** |  | .01 | .06 | **.22** |
| Text NC | .11 | **.26** | **.29** |  | .08 | .16 | **.25** |  | .09 | **.19** | **.28** |  | **.21** | **.38** | **.48** |
| All texts | **.24** | **.34** | **.38** |  | .09 | **.25** | **.31** |  | **.20** | **.28** | **.37** |  | **.30** | **.41** | **.48** |

*Note*. Bold figures indicate *p* < .05. DA (Discomfort with Ambiguity), AB (Absolutism), and NC (Need for Complexity and Novelty).
